# Fisheries shocks provide an opportunity to reveal multiple recruitment sources of sardine in the Sea of Japan

**DOI:** 10.1038/s41598-024-72925-8

**Published:** 2024-09-17

**Authors:** Tatsuya Sakamoto, Motomitsu Takahashi, Kotaro Shirai, Tomoya Aono, Toyoho Ishimura

**Affiliations:** 1 Fisheries Resource Research Institute, Japan Fisheries Research and Education Agency, Nagasaki, Japan; 2 Atmosphere and Ocean Research Institute, The University of Tokyo, Chiba, Japan; 3 Department of Chemistry and Material Engineering, National Institute of Technology, Ibaraki College, Ibaraki, Japan; 4 Graduate School of Human and Environmental Studies, Kyoto University, Kyoto, Japan; 5 Hakubi Center, Kyoto University, Yoshidanihonmatsucho, Kyoto Sakyo-ku, Kyoto, 606-8316 Japan

**Keywords:** Migration, Otolith isotopes, Population structure, Recruitment, Sardine, Source-sink dynamics, Animal migration, Biooceanography, Conservation biology, Population dynamics, Stable isotope analysis, Marine biology

## Abstract

The abrupt decline in sardine catches in the Sea of Japan and the East China Sea (SJ-ECS) in 2014 and 2019 and the recovery in the following years call into question the current assumption that sardines in the SJ-ECS form a self-recruiting subpopulation. To test this hypothesis, we analysed otolith stable oxygen and carbon isotope profiles (*δ*^18^O, *δ*^13^C) of age-0 and age-1 sardines from 2010 and 2013–2015 year-classes captured in the SJ-ECS, as geographic markers for nursery areas. Age-0 sardines generally showed a significant ontogenetic decrease in otolith *δ*^18^O from larval to juvenile stages. However, the majority of age-1 captured in spring 2011, 2015 and 2016 showed non-decreasing otolith *δ*^18^O profiles, suggesting that the age-0 off the Japanese coast were not the main source of recruitment. Different migration groups were thus indicated: the “locals” growing up off the Japanese coast and the migrating “nonlocals”. The isotope profiles of the “nonlocals” overlapped with those of age-0 captured in the subarctic North Pacific, suggesting that they may be migrants from the Pacific, or perhaps an unobserved northward migration group in the SJ-ECS. Our results highlight the considerable uncertainty in the population structure assumed in current stock assessment models for Japanese sardine.

**Supplementary Information:**

The online version contains supplementary material available at 10.1038/s41598-024-72925-8.

## Introduction

Understanding the seasonal movements and origins of exploited marine fish is crucial for assessing population linkages and defining management unit (i.e. stock) boundaries. Biomass estimations often rely on the assumption that the unit consists of fish with uniform vital rates (e.g., growth, mortality), and that the available data (e.g., observed catch, abundance indices, size or age composition) reflect recruitment within the stock rather than immigration from neighbouring units^1^. Disregarding the mixing of recruits from different origins and life-history traits may therefore compromise the accuracy of population productivity estimates^2,3^ and hinder downstream studies, e.g. on the causes of biomass fluctuations, which are needed for future projections.

**Fig. 1:**
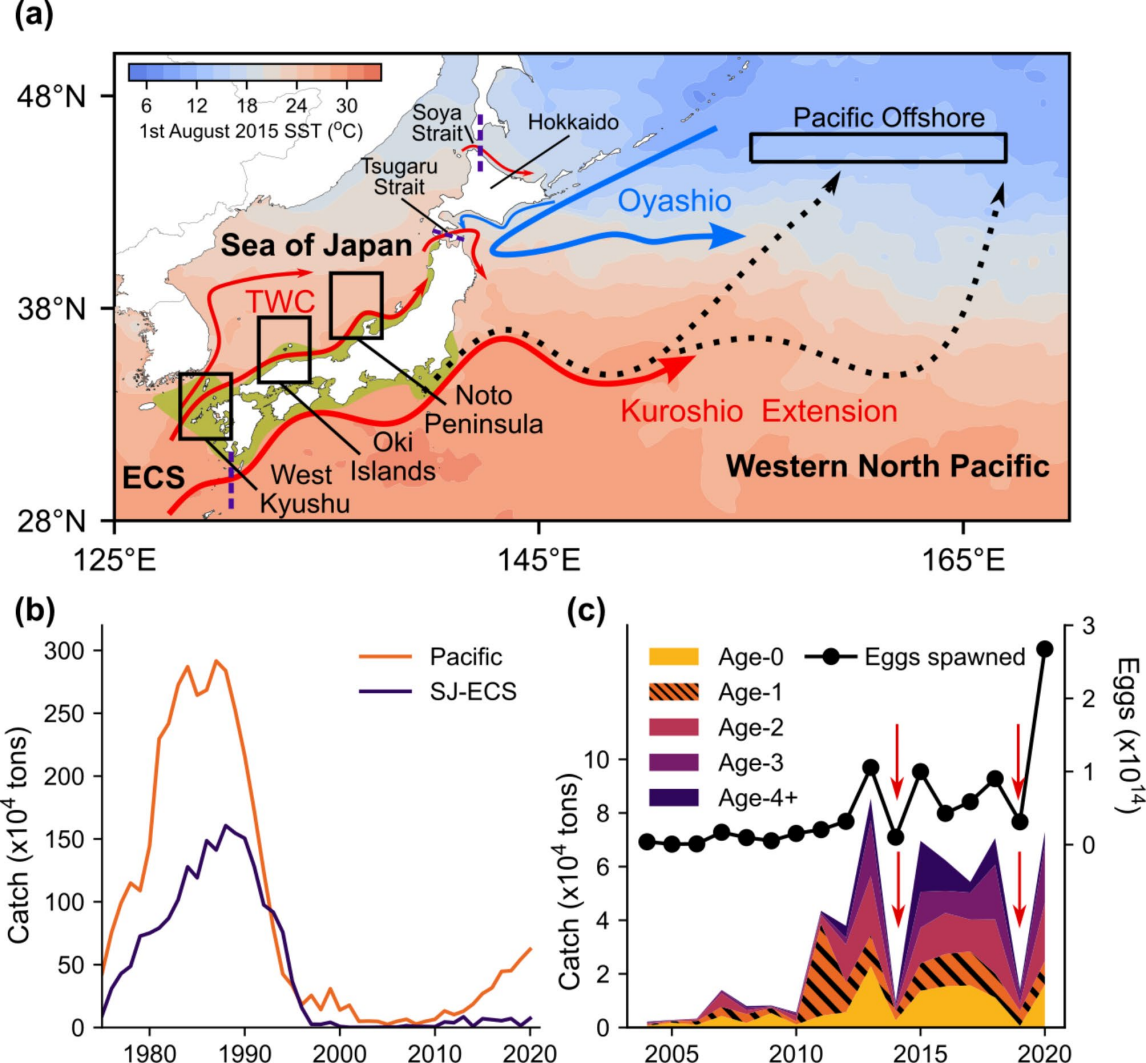
(**a**) Schematics of ocean currents around distribution of Japanese sardine together with sample collection sites (black squares), spawning grounds (yellow shades) and satellite-based sea surface temperature on 1st August 2015 based on Donlon et al.^49^. Red arrows show typical positions of warmer currents originating from subtropical Pacific and blue lines indicate cooler currents from subarctic regions. Black dotted lines indicate typical movement path of larvae and juveniles that use offshore nursery area in the Pacific. Purple dashed lines show boundaries of currently assumed two subpopulations, namely Tsushima Warm Current and Pacific subpopulation. (**b**) Long-term time series of Japanese fishery catch in Pacific and SJ-ECS based on Furuichi et al.^19^ and Muko et al.^20^. (**c**) Recent time series of egg abundance based on field surveys and catch of the sardine in SJ-ECS decomposed by age based on Muko et al.^20^. Red arrows indicate years of abrupt decline.

Sardines (*Sardinops* and *Sardina* spp.), globally distributed in temperate regions^4^, play a key role in energy transfer from planktons to higher trophic levels in productive marine ecosystems^5^ and are of great economic importance^6^. The western North Pacific and its marginal seas support one of the largest sardine populations on earth^4^ (Japanese sardine *Sardinops sagax melanostictus*), with annual catch exceeding 5 million tonnes in the late 1980s^7^. The population structure of Japanese sardine has been debated since at least the 1930s^8^. Some biological approaches have been applied to infer origins and movements of sardine, such as differences in the number of vertebrae^9^ or mitochondrial DNA^10^, which have generally failed to detect clear population structures. However, given the distribution of spawning grounds and the narrow straits separating the Sea of Japan and the North Pacific (Fig. 1a), current fisheries management assumes the existence of two semi-discrete subpopulations, the Tsushima Warm Current subpopulation distributed in the Sea of Japan and the adjacent East China Sea (hereafter the SJ-ECS) and the Pacific subpopulation in the western North Pacific (Fig. 1a), and treats them as management units.

These two sardine subpopulations are assumed to have their own main source of recruitment. Spawning grounds are formed from winter to spring in the inshore of the Kuroshio and Tsushima Warm Currents^11,12^ (Fig. 1a). The peak of spawning in the SJ-ECS occurs from March to May^12^. During summer, larvae and juveniles are widely distributed off the Japanese coasts and in the southern Sea of Japan near the spawning grounds^8,13,14^, and also in the offshore Kuroshio-Oyashio transition zone^15^ (Fig. 1a). Those that grew in coastal areas in the SJ-ECS are considered to be the main recruits of the Tsushima Warm Current subpopulation. Eggs and age-0 juveniles were hardly found off the North Korean or Russian coasts in the Sea of Japan during surveys and fisheries in the 1930s–40s and 1970s–80s^8,16^. For the Pacific subpopulation, juveniles distributed in the Kuroshio-Oyashio transition zone in summer and migrating northwards to the subarctic region^17^ are considered to be the main source of recruitment in recent years^15^. These recruits mature sexually at age one or two^18^, and migrate to coastal areas for reproduction during winter to spring.

However, some observations in the SJ-ECS cannot be explained by the current hypothesis of population structure. Time series of fisheries catches in the SJ-ECS and the Pacific, which are assumed to be from semi-independent subpopulations, show similar decadal trends: both peak in the late 1980s, collapse in the 1990s and show signs of recovery in the 2010s (Fig. 1b). Recent increases in sardine catch in the SJ-ECS began with the sudden appearance of age-1 fish in 2011, although few age-0 fish were caught in the system in 2010 (Fig. 1c). Coincidentally or not, an extremely strong year-class was produced in the Pacific in 2010^19^. Furthermore, sardine schools were hardly observed in the coastal areas of the SJ-ECS in spring 2014 and 2019. Sardine catches in the areas by Japanese vessels abruptly decreased to 10–30% in 2014 and 2019 compared to the prior years (Fig. 1c), as did catches by South Korean vessels^7^, suggesting that sardines were sparse in the SJ-ECS (Fig. 1a). Numbers of eggs and larvae in spawning surveys and catches of age-0 juveniles in Japanese coastal areas during summer to autumn also declined in these years^20^ (Fig. 1c, Supplementary Fig. S1). Fortunately, sardine schools returned to Japanese coasts in spring in the following years (2015 and 2020). Nevertheless, despite the likely limited reproduction in 2014 and 2019 in the SJ-ECS, age-1 fish hatched in 2014 and 2019 were present in the 2015 and 2020 catches in proportions comparable to other common years (Fig. 1c). These observations led us to question the origin of the recruits: are eggs, larvae and juveniles in the SJ-ECS the only source of recruits for the Tsushima Warm Current subpopulation?

The origin and nursery grounds of fish can be inferred from isotopic signatures in otoliths, the calcium carbonate formed in the inner ear (e.g. Rooker et al.^21^). The stable oxygen isotope value (*δ*^18^O) of fish otoliths is influenced negatively by temperature (for Japanese sardine, Sakamoto et al.^22^) and positively by seawater *δ*^18^O, and seawater *δ*^18^O is strongly correlated with salinity^23^. The stable carbon isotope value (*δ*^13^C) of the otolith reflects the isotope of two sources: dissolved inorganic carbon from the ambient water and metabolic carbon released from the respiration of food^24^. These values may therefore differ among fish from different regions, allowing discrimination of nursery areas^21,25^ and migration routes^17,26^. Recently, Aono et al.^14^ found that otolith *δ*^18^O profiles of age-0 sardines captured in the SJ-ECS in late summer consistently show marked decreasing trends, reflecting the significant seasonal warming of the region (Supplementary Fig. S2). Significant deviations from such trends may thus indicate different distributions in the first year of life.

We aimed to identify the recruitment sources of sardine in the SJ-ECS. To this end, otolith isotopes were analysed from age-0 and age-1 of 2010 and 2013–2015 year-classes of sardine caught in the SJ-ECS and western North Pacific during summer to autumn and the following spring, respectively (Fig. 1a). The analysis included the key 2010 and 2014 year-classes, which were characterised by a moderate or high catch at age-1, even though the egg abundance and catch of age-0 in the SJ-ECS were limited in the year of hatching (Fig. 1c). If juveniles that grew near the Japanese coast in the SJ-ECS are the main source of recruitment, as conventionally assumed, the otolith signatures of age-0 and age-1 fish should be similar, but this was not the case.

## Materials and methods

### Ethics declaration

No use of live animals was required for this study. All samples used for the present study came from animals fished for commercial purposes or the archived collections from the trawl surveys aimed for assessing recruitment of pelagic fish conducted by the Fisheries Research Institute, Japan Fisheries and Education Agency. The surveys have been approved by the Fisheries Agency, Japan and carried out with the certification for permitting the trawling written in Japanese. All procedures for fish sampling in were conducted in compliance with the “*Guidelines for handling live fish at FRI*” of the Fisheries Research Institute, Japan Fisheries and Education Agency (FRI), and with recommendations of the ARRIVE Guideline^27^.

### Otolith sample collection

To represent the entire nursery grounds of sardine in the SJ-ECS, otolith samples were collected from sardines captured in 2010–2011 and 2013–2016 in three major fishing areas, namely the regions around West Kyushu, the Oki Islands and the Noto Peninsula (Fig. 1a). The fish were captured in purse-seiner or set net fisheries or in midwater trawls during cruise surveys. Fish < 15 cm standard length (SL) captured during July to December were considered age-0 fish, and those < 16 cm SL captured during January to June were age-1 fish. To represent age-0 and 1 fish of the 2010 and 2013–2015 year-classes in each region, 2–8 individuals per sampling batch were selected from 1 to 3 sampling batches, except for age-0 of the 2010 and 2014 year-classes around the Noto Peninsula, as catch there was very low (Table 1). The 2015 year-class around the Oki Islands was sampled more frequently from September 2015 to May 2016 to observe seasonal variations in the proportions of different recruitment sources. Collected fish were frozen after landing or on board in − 20 °C, and thawed at a laboratory. After measurements of length and weight, the otoliths (sagittae) were extracted. The otoliths were cleaned using a thin brush and rinsed with fresh water.

**Table 1:** Metadata of collected samples from each region (*includes data from Aono et al.^14^, **includes unpublished data, ***all data from Sakamoto et al.^31^).

Region	Year-class	Age	Collection dates	SL (mm)	*N* (0–60 dph)	*N* (106–120 dph)
West Kyushu	2010	0	30 Aug, 31 Aug 2010	125.4 ± 6.5	10	10
	2013	0	13 Sep, 4 Oct 2013	139.8 ± 5.6	12	12
	2014	0	25 Aug, 2014	132.8 ± 7.3	6	6
	2015	0	2 Sep, 12 Sep 2015	118.8 ± 5.5	6*	6*
	2010	1	11 Jan, 2011	147.8 ± 1.9	6	6
	2013	1	19 Jan, 25 Feb, 13 Mar 2014	149.1 ± 2.6	19	12
	2014	1	29 Jan, 13 Feb, 17 Mar 2015	152.2 ± 2.3	17**	5**
	2015	1	17 Jan, 2016	138.2 ± 5.9	12	3
Oki Islands	2010	0	14 Jul, 2010	97.9 ± 4.0	6	6
	2013	0	3 Sep, 22 Nov, 12 Dec 2013	134.1 ± 8.5	22	19
	2014	0	26 Aug, 8 Oct 2014	124.2 ± 9.7	15	11
	2015	0	1 Sep, 5 Nov, 1 Dec 2015	127 ± 13.0	21**	10**
	2010	1	22 Apr, 13 May 2011	135.9 ± 10.2	14	14
	2013	1	7 Apr, 20 May 2014	149.2 ± 6.0	16	8
	2014	1	24 Feb, 25 Feb 2015	143.9 ± 6.2	17**	6**
	2015	1	18 Feb, 8, 9,14 Mar, 12 Apr, 19, 20, 24, 30 May 2016	146.5 ± 5.0	35	33
Noto Peninsula	2010	0	–	–	0	0
	2013	0	11 Jul, 2 Sep, 12 Sep 2013	85.8 ± 13.5	25	12
	2014	0	–	–	0	0
	2015	0	23 Aug, 27 Aug, 8 Sep 2015	93.2 ± 15.3	20*,**	13*,**
	2010	1	17 Mar, 11 May 2011	132.5 ± 3.9	16	16
	2013	1	21 Apr, 25 Apr, 29 May 2014	140.9 ± 3.8	22	11
	2014	1	16 Apr, 23 Apr, 8 May 2015	142.3 ± 4.9	30**	15**
	2015	1	10 Mar, 11 Apr 2016	144.4 ± 12	11	4
Pacific Offshore	2010	0	28 Sep, 1, 2, 4, 7 Oct 2010	115.5 ± 5.7	25***	25***
	2013	0	–	–	0	0
	2014	0	23 Sep, 24 Sep 2014	130.1 ± 4.1	29***	29***
	2015	0	18 Sep, 19 Sep, 20 Sep, 21 Sep 2015	131.1 ± 5.7	30***	30***
				(Total)	442	322

### Otolith processing, microstructure and isotope analyses

Otoliths were embedded in epoxy resin (Petropoxy 154, Burnham Petrographics LLC), then polished along the sagittal plane until the core is revealed using sandpapers and alumina suspension (BAIKOWSKI International Corporation). The daily increments were examined along the axis in the postrostrum from the core as far as possible using an otolith measurement system (RATOC System Engineering Co. Ltd.). For 322 otoliths, the otolith portion formed during 0–60 dph, representing the larval stage, was identified and milled out using a high-precision micro-milling system Geomill 326 (Izumo-web, Japan). For 215 otoliths, otolith portion formed during 106–120 dph was additionally milled out to represent values for the juvenile stage. The milling depth for the 0–60 dph and 106–120 dph portions was 50 and 100 μm, respectively. The *δ*^18^O and *δ*^13^C of powdered samples were analysed using an isotope ratio mass spectrometer (Delta V plus, Thermo Fisher Scientific) equipped with an automated carbonate reaction device (GasBench II, Thermo Fisher Scientific) at the Atmosphere and Ocean Research Institute, the University of Tokyo, Chiba^28^. The otolith powder (8 to 80 µg) was reacted with phosphoric acid at 72 °C. All isotope values are reported using delta notation relative to the Pee Dee Belemnite. Analytical precisions of *δ*^18^O and *δ*^13^C for international standards (NBS-19) were 0.06–0.13‰ (1σ) and 0.05–0.11‰, respectively. The commonly accepted acid fractionation factor of 1.01025 for calcite^29^ was used. Because the difference between the acid fractionation factor of calcite (standard material) and aragonite (otolith) depends on temperature^30^, we subtracted 0.09‰ from the *δ*^18^O value to allow comparison with data in previous studies analysed at 25 °C (e.g., Sakamoto et al.^22^).

To increase data coverage, unpublished data for otoliths of 6 age-0 and 22 age-1 fish captured in 2015, and published data from Aono et al.^14^ for age-0 in SJ-ECS and Sakamoto et al.^31^ for age-0 in the Pacific were added to the dataset (Table 1). For the unpublished data, microstructure analysis was not performed for the otoliths of the 22 age-1 individuals. The profiles had the resolution of 5–30 days or 30–160 μm, and the *δ*^18^O were determined by a customized continuous-flow isotope ratio mass spectrometry system (MICAL3c with IsoPrime100) at the National Institute of Technology, Ibaraki College, Hitachinaka, Japan^32,33^. The otolith powder (0.3 to 5.5 µg) was reacted with phosphoric acid at 25 °C, and the analytical precisions were within ± 0.1‰ for both *δ*^18^O and *δ*^13^C. For otoliths for which microstructure analysis was not performed, corresponding age range for each milling area was later estimated from distance from the core using the mean relationship between otolith radius and age of other fish captured in the same year, season and region. As the additional unpublished and published data had higher resolution, data were rescaled to 0–60 dph and 106–120 dph resolution. Here, the *δ*^18^O and *δ*^13^C values of which median of corresponding age range falls in 0–60 and 106–120 dph were averaged, linearly weighted by the width of milling area.

### Definitions of the “locals”, “nonlocals” and “Pacific-offshores”

Aono et al.^14^ found that otolith *δ*^18^O profiles of age-0 sardines captured in the SJ-ECS consistently show marked ontogenetic decreases during larval to juvenile stages, which was indeed the trend for the age-0 fish in the SJ-ECS and age-1 around West Kyushu in this study. Significant deviations from such trends are indicative of a different nursery area. The individuals captured in the SJ-ECS were therefore split into two groups, the “locals” which likely grew up in the SJ-ECS and the “nonlocals” which potentially did not, based on the otolith *δ*^18^O for the juvenile stage (106–120 dph). For each year-class, the highest otolith *δ*^18^O for the juvenile stage of the age-0 sardine in the SJ-ECS and age-1 around the West Kyushu were defined as the threshold, and the individuals that had a lower or equal otolith δ^18^O for the juvenile stage than the threshold were designated as “locals” and those with a higher value as “nonlocals”. The age-0 in the SJ-ECS without the measurement of otolith *δ*^18^O for the juvenile stage was also assigned to the “locals” as they likely grew up in the SJ-ECS. Age-1 around the Oki Islands and the Noto Peninsula lacking otolith *δ*^18^O for the juvenile stage were later categorized as either “locals” or “nonlocals” using a linear discriminant analysis based on the values for larval stage (see below). Age-0 sardines collected in the subarctic offshore region in the North Pacific were defined as “Pacific-offshores”.

### Discrimination of the locals and nonlocals

To understand the differences in early life-history traits among locals, nonlocals and Pacific-offshores, differences in otolith *δ*^18^O and *δ*^13^C for 0–60 dph and otolith radius at 60 dph were tested using a multivariate analysis of variance (MANOVA). Data of locals and nonlocals of all 4 year-classes were pooled for MANOVA, although the individuals that lacked measurement of the otolith *δ*^18^O for the juvenile stage or the daily increments were excluded. Tests for univariate and multivariate normalities, multicollinearity, linearity, homogeneity of covariances and variance were performed before MANOVA (see Supplementary Materials S1 and Methods for details and their results). The non-parametric Kruskal–Wallis test and Games–Howell test were used for post-hoc tests and pairwise comparisons.

A linear discriminant function analysis was performed to classify age-1 fish whose otolith *δ*^18^O for the juvenile stage was not analysed. Using otolith *δ*^18^O and *δ*^13^C for larval stage and otolith radius at 60 dph of locals and nonlocals of all year-classes as learning data, a linear discriminant function was developed and applied to unknown age-1 data to predict the most likely classification. The accuracies of the prediction models were estimated by leave-one-out cross-validation. These analyses were performed using Python 3.8.8 with Scikit-learn 0.24.1 library^34^.

### Prediction of potential nursery areas of the nonlocals

To explore the likely migration pattern of the nonlocals, possible distributions during the larval and juvenile stages were inferred from comparison between the predicted isoscape and observed otolith *δ*^18^O (Supplementary Methods). As the distributions may be either within the SJ-ECS or in the western North Pacific, we made inferences for both possibilities. Briefly, the isoscapes of otolith *δ*^18^O for 0–60 dph and 106–120 dph were estimated based on mean temperature and salinity distributions at 10 m depth during 0–60 and 106–120 days from the assumed hatch dates (every 3 days between mid-April and mid-May, Supplementary Fig. S1) and empirical relationships between otolith *δ*^18^O and temperature and *δ*^18^O of seawater and seawater *δ*^18^O and salinity. The temperature and salinity distributions were obtained from data-assimilated hydrodynamic models for each region^35,36^. The model grid points whose predicted otolith *δ*^18^O was within the range of the mean ± 1 standard deviation (SD) of the otolith *δ*^18^O of nonlocals of each year-class were considered as potential distributions. The hatch dates were assumed based on the observations that spawning during 2013–2015 in the SJ-ECS peaked during April to May (Supplementary Fig. S1), and most frequently occurred hatch dates of juveniles found in the Kuroshio–Oyashio transition zone were also between mid-April and mid-May^15^. To consider the possibility that the nonlocals hatched during marginal spawning months in the SJ-ECS, the distributions were also predicted assuming the hatch dates in February and in June. See Supplementary Materials and methods and Supplementary Fig. S3 for further details and accuracy assessments of the hydrodynamic models.

## Results

### Otolith *δ*^18^O profiles

**Fig. 2:**
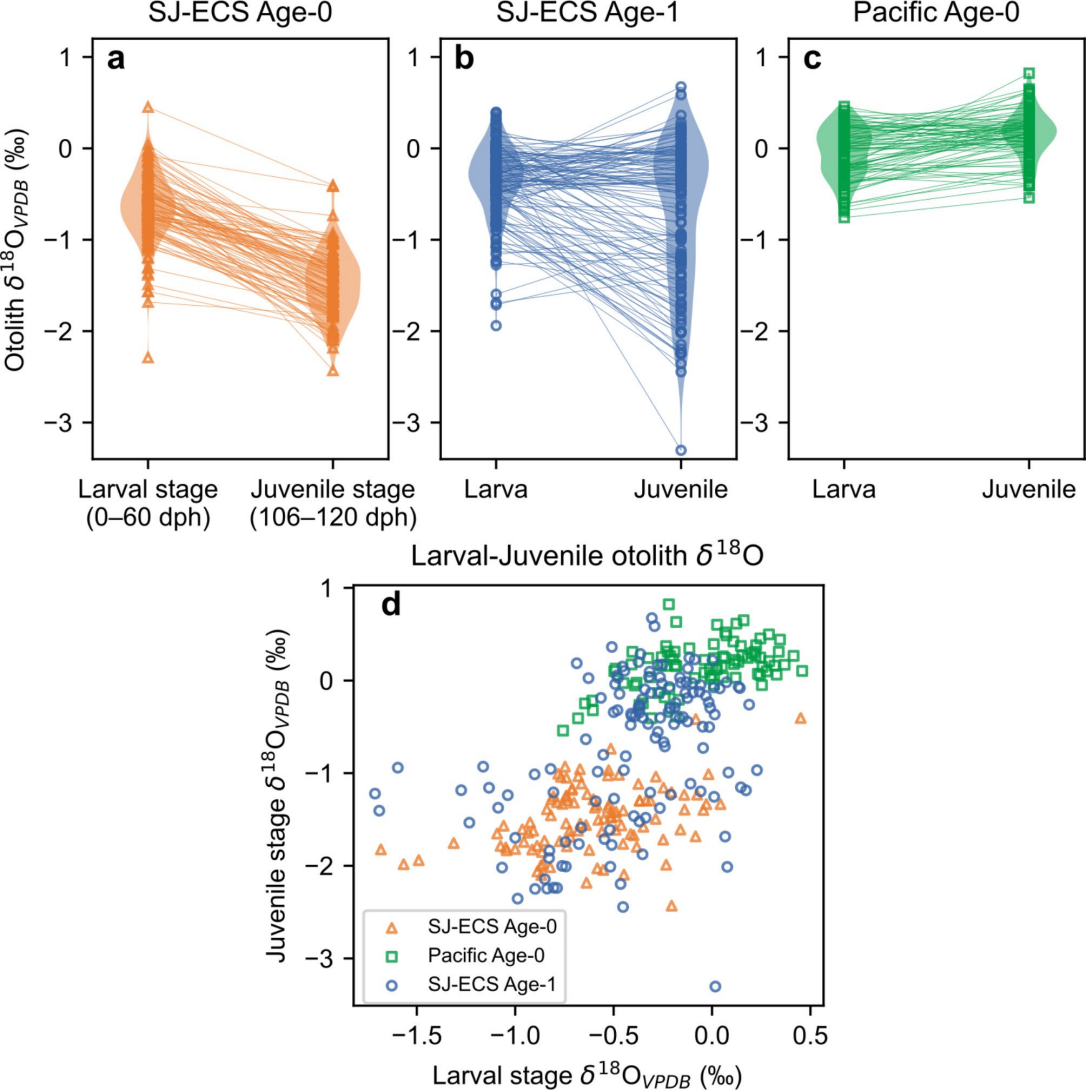
Otolith *δ*^18^O profiles (larval stage: 0–60 dph, juvenile stage: 106–120 dph) of age-0 (**a**) and age-1 (**b**) sardines captured in the Sea of Japan and East China Sea, and age-0 fish from the subarctic offshore area in the Pacific (**c**). Data for all year-classes analysed are pooled and plotted with violin plot representing data density. Spring and summer otolith *δ*^18^O values for each individual are also shown as a scatter plot (**d**).

**Fig. 3:**
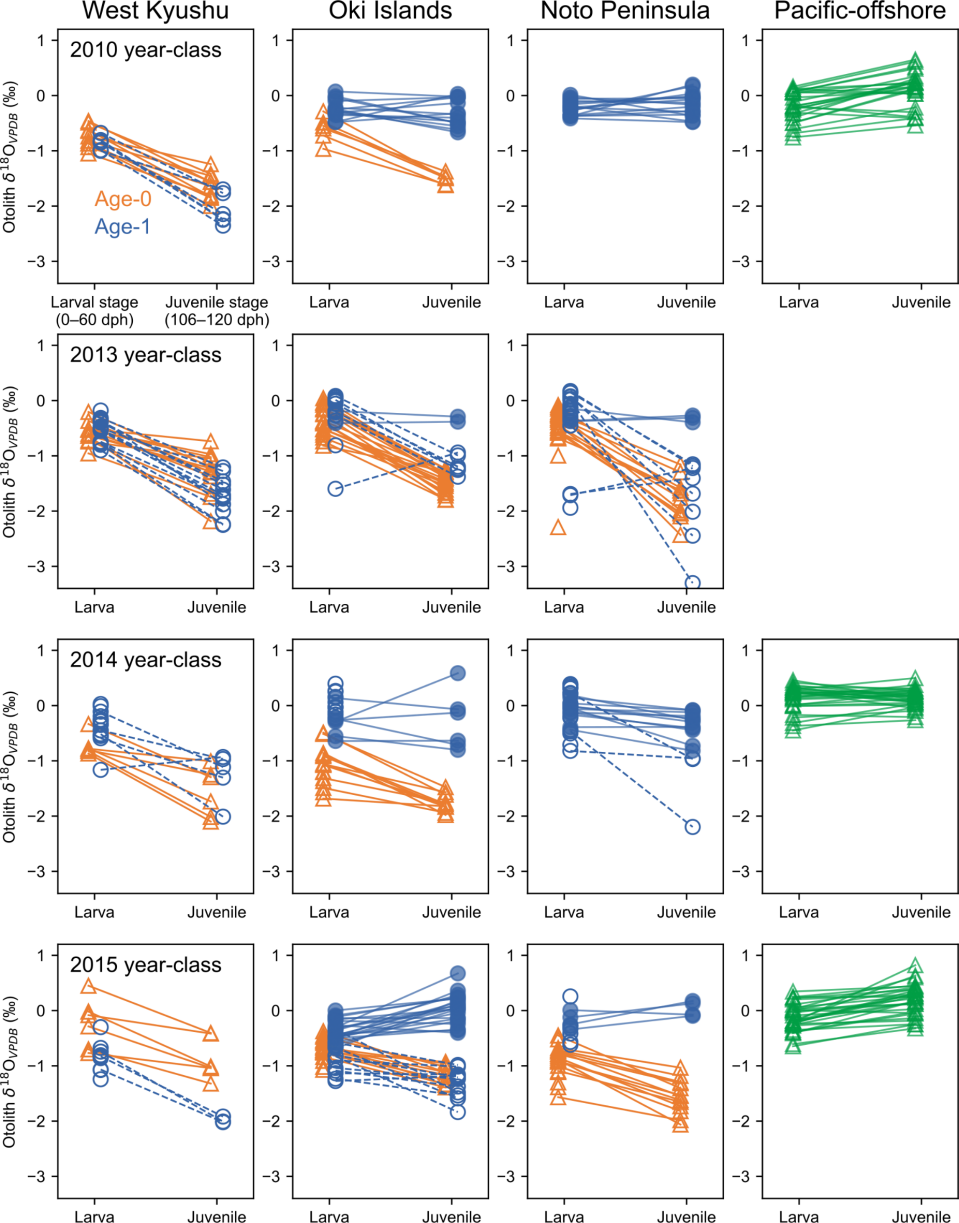
Ontogenetic changes of otolith *δ*^18^O in age-0 (orange) and age-1 (blue) sardines captured around West Kyushu (first column), Oki Islands (second column), Noto Peninsula (third column) and Pacific offshore (fourth column). Data for 2010, 2013, 2014 and 2015 year-classes are presented in each row. Data of age-1 (blue) defined as locals is shown in open circles and dotted lines and that of nonlocals is shown in filled circles with solid lines. Data for age-0 is shown in triangles with solid lines.

Otolith *δ*^18^O of the age-0 fish captured in the SJ-ECS (around West Kyushu, the Oki Islands and the Noto Peninsula) during 2010 and 2013–2015 ontogenetically decreased from − 0.7 ± 0.4‰ (mean ± 1 SD) for larval stage (0–60 dph) to − 1.5 ± 0.4‰ for juvenile stage (106–120 dph) (the locals, Fig. 2a). This is consistent with the negative correlation between otolith *δ*^18^O and temperature^22^ and the significant seasonal warming in the SJ-ECS (Supplementary Fig. S2). However, while some of the age-1 fish in the SJ-ECS showed similarly low *δ*^18^O for juvenile stage (the locals), a number of age-1 fish in the SJ-ECS did not (the nonlocals, Fig. 2b). Otolith *δ*^18^O of age-0 from the offshore subarctic North Pacific (the Pacific-offshores) showed slight ontogenetic increases (larva: − 0.1 ± 0.3‰, juvenile: 0.1 ± 0.3‰, Fig. 2c). The otolith *δ*^18^O values for the juvenile stage were significantly lower in age-0 fish in SJ-ECS than in Pacific-offshores, and the values of age- 1 fish in SJ-ECS were distributed among both groups (Fig. 2d). For each year-class and sampling region analysed, almost all age-1 fish from West Kyushu showed a seasonal decrease in otolith *δ*^18^O (larva: − 0.6 ± 0.3‰, juvenile: − 1.7 ± 0.4‰, also categorised as locals, Fig. 3), and some age-1 fish from the Oki Islands and Noto Peninsula showed non-decreasing trends (the nonlocals, larva: − 0.3 ± 0.2‰, juvenile: − 0.2 ± 0.3‰, Fig. 3). Exceptionally low *δ*^18^O values for larval stage between − 2.5 and − 1.6‰ were observed from five individuals mostly captured in 2014, which were consistent with the reported values of larvae captured in the less-saline bay near Noto Peninsula^37^ (–2.4 to − 1.0‰).

### Otolith *δ*^18^O and *δ*^13^C and otolith radius for larval stage

**Fig. 4:**
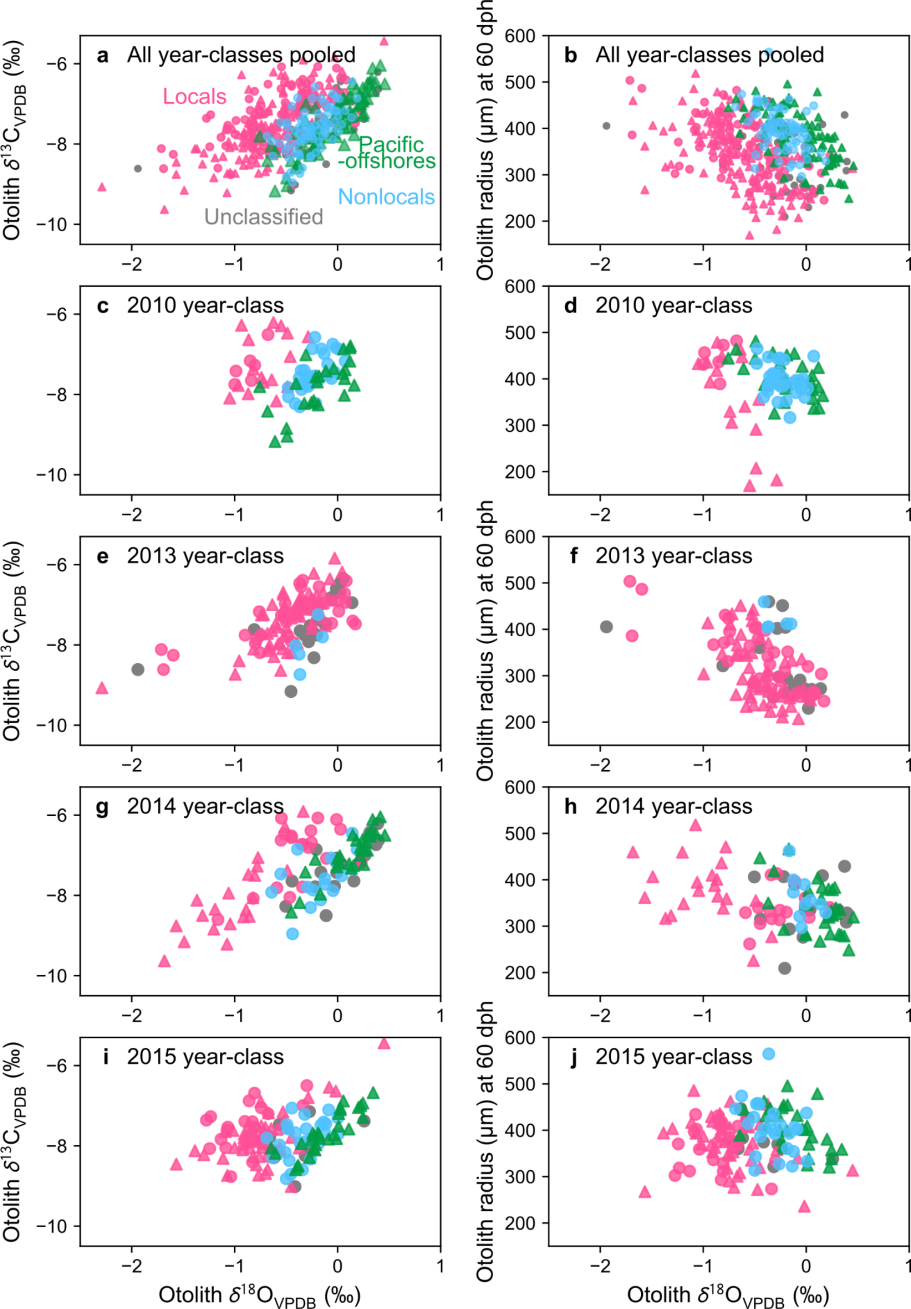
Relationships between otolith *δ*^18^O and *δ*^13^C for larval stage (0–60 dph, **a**,**c**,**e**,**g**,**i**) and between otolith *δ*^18^O for larval stage and otolith radius at 60 dph (**b**,**d**,**f**,**h**,**j**) for all year-classes pooled (**a**,**b**), 2010 year-class (**c**,**d**), 2013 year-class (**e**,**f**), 2014 year-class (**g**,**h**) and 2015 year-class (**i**,**j**). Locals are shown in pink, nonlocals in light blue and pacific-offshores in green. Gray plots are age-1 fish that are unclassified due to the lack of otolith *δ*^18^O for juvenile stage. Circles and triangles show age-1 and age-0 fish, respectively.

Otolith *δ*^18^O and *δ*^13^C values for larval stage and otolith radius at 60 dph of the locals, nonlocals and Pacific-offshores were significantly different between groups (Fig. 4a,b, MANOVA, F(6, 614) = 48.78, *p* < 2.2 × 10^−16^). Post-hoc Kruskal–Wallis test showed that *δ*^18^O and otolith radius had significant differences among groups (*δ*^18^O: Chi square = 122.0, *p* = 2.6 × 10^−27^, df = 2, *δ*^13^C: Chi square = 3.63, *p* = 0.16, df = 2, otolith radius: Chi square = 26.8, *p* = 1.6 × 10^−6^, df = 2). Pairwise comparisons using the Games–Howell test showed that nonlocals had significantly higher mean otolith *δ*^18^O and otolith radius at 60 dph than locals (adjusted *p* value < 8.9 × 10^−14^ and = 2.4 × 10^−8^, respectively, Supplementary Table S1), and lower mean otolith *δ*^18^O than the Pacific-offshores (adjusted *p* value = 7.6 × 10^−6^). These significant differences show that the nursery areas of locals and nonlocals are not common during the larval stage, and the nonlocals experienced cooler temperatures than the locals. Despite the significant difference in the mean otolith *δ*^18^O, the overall value ranges of the nonlocals consistently included those of the Pacific-offshores (Fig. 4a,b) in each year-class (Fig. 4c–j). Some Pacific-offshore individuals which had hatched earlier in the season (between 6 and 10 weeks after January 1st) had *δ*^18^O values for larval stage above 0.2‰, whereas the highest *δ*^18^O value observed in otoliths from non-locals was 0.2‰ (Supplementary Fig. S4).

Cross-validation of the linear discriminant function analysis of locals and nonlocals of all year-classes using the three variables correctly classified a total of 202 of 227 individuals (89%). Out of 153 individuals classified as locals, 142 (93%) were actually locals and out of 74 individuals classified as nonlocals, 60 (81%) were actually nonlocals. Based on the discriminant function, the 45 unclassified age-1 fish were divided into 20 locals and 25 nonlocals.

### Seasonal and inter-annual variation in locals/nonlocals proportions in the Sea of Japan

**Fig. 5:**
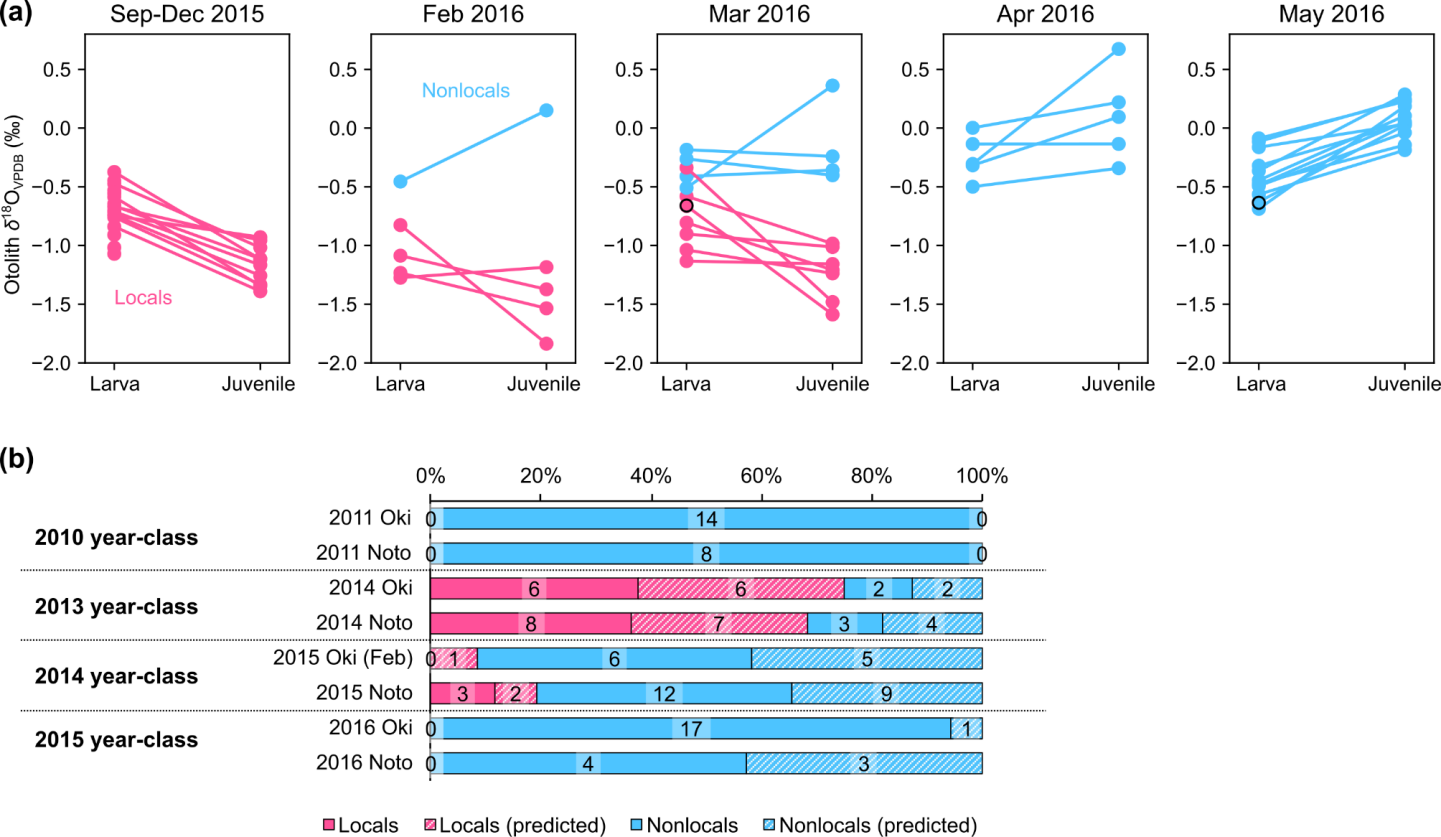
Otolith *δ*^18^O profiles of 2015 year-class repeatedly sampled from September 2015 to May 2016 around Oki Islands (**a**). Inter-annual variation of locals/nonlocals proportions in age-1 fish captured during spring (April to May, except for those around the Oki Islands 2015) (**b**). Numbers indicate number of individuals in each group. Data of individuals classified by discriminant analysis is shown in black edge plots (**a**) or in shaded bars (**b**).

To understand the timing of arrival of the nonlocals, 2015 year-class around the Oki Islands was repeatedly sampled from September 2015 to May 2016. From September to December 2015, all 21 fish in the 2015 year-class were locals by definition, and all 10 otolith *δ*^18^O values analysed for the juvenile stage were lower than − 0.9‰ (Fig. 5a). The proportion of the nonlocals increased towards spring in 2016: one in 5 (20%) in February, 4 in 12 (33%) in March, 5 in 5 (100%) in April and 13 in 13 (100%) in May were nonlocals (Fig. 5a). Thus, while the locals were dominant in 2015 autumn, the nonlocals increased from winter onwards and completely replaced the locals in spring.

The proportions of locals and nonlocals in April and May, the main spawning season in the Sea of Japan, showed consistent inter-annual fluctuations around the Oki Islands and Noto Peninsula (Fig. 5b). Note that because no age-1 samples were available from the Oki Islands in April and May 2015, the samples caught around the Oki Islands in February 2015 were used for comparison instead. As the locals/nonlocals proportions in individuals predicted by linear discriminant analysis were similar for individuals grouped based on otolith *δ*^18^O for the juvenile stage, we considered the predictions to be largely accurate. In 2011, 2015 and 2016, the nonlocals were the majority, with a proportion of 80–100% around both the Oki Islands and the Noto Peninsula (Fig. 5b). Additionally, the 8 age-1 individuals captured in March 2011 around Noto Peninsula were all nonlocals (Fig. 3, not shown in Fig. 5). These suggest that it is the nonlocals that move around the Sea of Japan in winter to spring along with the schools of spawning adult fish, and therefore mainly recruit there. Only in 2014, when total sardine catch in SJ-ECS decreased significantly (Fig. 1b), locals constituted the majority around both the Oki Islands and the Noto Peninsula, at 75% and 73% respectively, and nonlocals were not dominant.

### Potential nursery areas of nonlocals during larval and juvenile stages

**Fig. 6:**
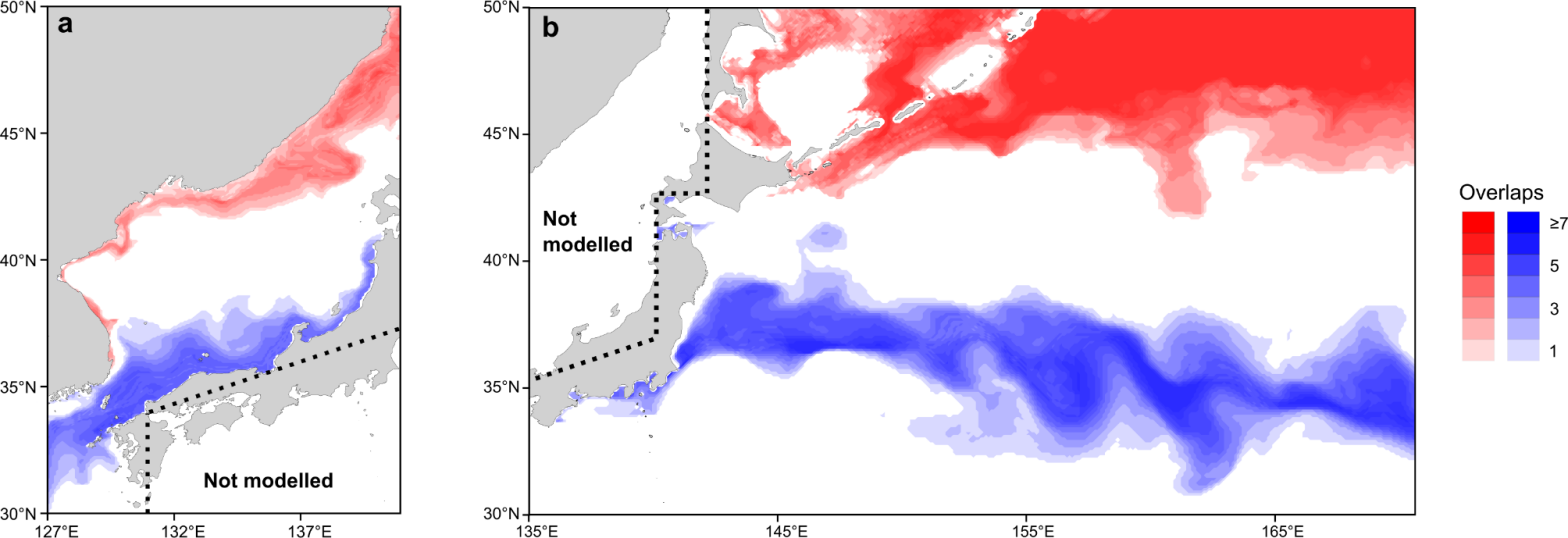
Potential distribution of nonlocals of 2015 year-class during the larval (0–60 dph, blue) and juvenile (106–120 dph, red) stages in the SJ-ECS (**a**) and North Pacific (**b**) predicted based on otolith *δ*^18^O values and hydrodynamic models. The shades indicate the areas where the environmental conditions (temperature and salinity) would produce an otolith *δ*^18^O within 1 SD of the mean value for the nonlocals. Darker shades indicate the areas where the environmental conditions match for multiple hatch dates within the range tested (every 3 days between mid-April and mid-May). Dotted lines suggest the boundary of the modelled area. See Supplementary Information S1 for the predictions for 2013 and 2014 year-class, and those assuming hatch dates in February or June, the marginal spawning months.

To explore the hypothesis of the migration pattern of the nonlocals, we inferred their potential distributions during larval and juvenile stages based on the comparison of otolith *δ*^18^O values and isoscape predicted by hydrodynamic models (Fig. 6). The distribution of the nonlocals in 2015 year-class for larval stage (0–60 dph, of which median date corresponding to mid-May to mid-June assuming hatch dates as mid-April to mid-May) was predicted to be either the southern coastal areas in the Sea of Japan (Fig. 6a) or the offshore area along the Kuroshio Extension in the North Pacific (Fig. 6b). For juvenile stage (106–120 dph, corresponding to late July to late August), the predicted distributions shifted northward to the northern coastal areas of the Sea of Japan (Fig. 6a) or the subarctic Oyashio region north of 42°N (Fig. 6b). The predicted patterns were similar for the nonlocals of the 2013 and 2014 year-classes (Supplementary Fig. S5). When distributions were predicted assuming that they hatched in the SJ-ECS during February or June, the marginal spawning months, the predicted distribution for the larval stage shifted either considerably southwards or northwards, or to the small inner bay (Supplementary Fig. S6). In the projections assuming hatch month in June (Supplementary Fig. S6d–f), the larval distributions were far away from the spawning ground where eggs and early-stage larvae have not previously been recorded and that are outside of the area covered by larval surveys (Supplementary Fig. S1).

## Discussion

In this study, we examined the stable isotopes of the otoliths of age-0 and age-1 Japanese sardine captured in the SJ-ECS to test the conventional hypothesis of self-recruitment. Contrary to the hypothesis, the locals that grew up in the Japanese coastal areas of the SJ-ECS were not the primary source of recruitment, with greater contributions from the nonlocals that have considerably different otolith isotope values. One might presume that the nonlocals are later-hatched cohorts (e.g., in June) of the locals that were too small to be caught in the surveys or fisheries during summer to autumn. However, the proportion of eggs spawned in marginal spawning months are usually insignificant [2–4% in February and < 1% in June in 2013 and 2015 (Fisheries Research and Education Agency, unpublished data)], and the nonlocals experienced cooler temperatures than the locals during the larval stage (Fig. 3; Table 1) that are rather indicative of hatching earlier. Could they be the earlier-hatched cohort (e.g., in February)? They would then experience significant seasonal warming (Supplementary Fig. S2) and show a marked ontogenetic decrease in otolith *δ*^18^O, which was indeed the case for February-hatched individuals in 2016 year-class^14^. We therefore consider that the nonlocals are unlikely to be part of locals that hatched outside the main spawning season, and more likely to be a group with a different migration pattern. Relatedly, the marked decrease of the proportion of the nonlocals in the 2014 spring suggests that the abrupt decrease of catch in 2014 is likely a consequence of the change in the migration pattern of the nonlocals and accompanying adults (Fig. 5b).

The key question is where the nonlocals came from. Because the nonlocals were found from the Sea of Japan in spring but not from the West Kyushu in the East China Sea in winter (Fig. 3), nonlocals were likely distributed somewhere north of the coastal areas during summer to autumn and migrated to the southern Sea of Japan during winter. Predictions of possible distribution provided main two hypotheses for movement patterns from the larval to juvenile stages (Fig. 6a,b), namely from the southern coastal areas of the SJ-ECS to the northern coastal areas of the Sea of Japan, and from the Kuroshio Extension area to the subarctic Oyashio area in the western North Pacific. The first pattern is consistent with the hypothesis that eggs and larvae in the southwest Sea of Japan may have been transported to the offshore area by the offshore branch of the Tsushima Warm Current in 1970–80s^38^. Sardines tend to expand their distribution with population growth^39^, and adults were abundant in the northern Sea of Japan in summer in 1930–40s and 1970–80s when the biomass was high^8,16,38^. Meanwhile, in the limited data from historical surveys and fisheries in the northern Sea of Japan, sardine eggs, larvae and age-0 fish were hardly caught even in 1930–40s and 1970–80s^8,16^. In addition, if the nonlocals originated from the southern Sea of Japan or the East China Sea (Fig. 6a), the abundance or proportion of the age-1 nonlocals would likely decrease in spring 2015 due to the severely limited spawning off the Japanese coast in 2014 (Fig. 1b), but this was not the case (Fig. 5b).

We then consider the possibility that the nonlocals originated from the western North Pacific. The idea of sardine migration from the Pacific was originally put forward by Nakai^8^, who found that sardine catches per unit effort off the Korean peninsula were strongly correlated with catches off Hokkaido in the previous year (fished mainly on the Pacific side, see Fig. 1a) between 1929 and 1941. Baba^40^ also mentioned the possibility of mixing based on the detection of infections of the parasite *Anisakis simplex* sensu stricto on adult sardines caught in SJ-ECS in February and March, which is usually prevalent in the western North Pacific and not in SJ-ECS. Some pelagic species are already known to migrate into the Sea of Japan against the strong current towards the Pacific in the Tsugaru Strait (e.g., Japanese common squid *Todarodes pacificus* (e.g., Sakaguchi^41^), Masu salmon *Oncorhynchus masou* (e.g., Sato & Shibuya^42^). The overlapping otolith isotopes and growths of the nonlocals and the Pacific-offshores (Figs. 2, 3 and 4; Supplementary Fig. S4) are consistent with these observations. In addition, the time series of recruitment and recruits per spawner estimated by current stock assessment models for the Pacific and Tsushima Warm Current stocks showed significantly similar fluctuations (Supplementary Fig. S7; year-to-year differences in recruitment: Pearson’s *r* = 0.87, *p* = 6.7 × 10^−15^, in recruits per spawner: *r* = 0.49, *p* = 5.7 × 10^−4^), indicating that the main recruitment sources of the two stocks are common. Why do the decadal variations in catches in the Pacific and the SJ-ECS synchronise, and why can age-1 fish be abundant in the SJ-ECS, as in 2011, 2015 and 2020, even though egg production and age-0 catches were severely limited in the previous year? These phenomena are clearly explained if the recruits in the SJ-ECS are mainly migrating from the Pacific.

Our results provide important implications for fisheries management and developments in stock assessment for Japanese sardine. The current stock assessment for the sardine subpopulations is subject to considerable uncertainty, as it is based on an incomplete understanding of recruitment processes. As the Tsushima Warm Current subpopulation may not be closed, the estimate of recruitment can be significantly biased depending on the recruitment of the Pacific subpopulation, especially if the migrants subsequently spawn in the SJ-ECS. Considerations for a move to models that address mixing by incorporating empirically estimated mixing rates^3^ or even removing management unit boundaries need to be started. Nevertheless, we cannot and should not exclude the alternative possibilities that the nonlocals are a northward-migrating group within the Sea of Japan that has not yet been observed, or those from very different hatch seasons in the SJ-ECS. Therefore, we cannot emphasise enough the importance of further efforts to confirm the origin and migration pattern of the nonlocals, ideally in international collaboration, to improve fisheries management for this species. As the overlap in otolith chemical signatures can still indicate different origins in similar environments (Fig. 6a,b), extensive research using multidisciplinary approaches, including basic biological metrics^43^, genomics^44^, parasite load^40^, environmental DNA^45^, biophysical modelling^46^ and cruise surveys must be conducted.

Overall, the analyses of the otoliths revealed the complexity of the population structure of Japanese sardine. The abrupt declines in sardine catches in 2014 and 2019 not only motivated us to investigate the population structure, but also helped us do so by naturally acting as a control experiment, thereby highlighting the importance of collecting samples and data during anomalous years. Decline in fisheries catches often raises questions about assumptions in management strategies and prompt studies on the migratory ecology of species (e.g., Rooker et al.^21^; Neat et al.^47^). Accumulated knowledge about population connectivity can lead to a change in management settings, ultimately leading to fish biomass recovery and sustainable fisheries under science-based management^48^. A decline in catch, while not at all beneficial to fisheries in the short term, could lead to a healthier marine ecosystem with sustainable fisheries production if research communities respond correctly.

## Electronic supplementary material

Below is the link to the electronic supplementary material.

Supplementary Material 1

## Data Availability

The datasets generated and/or analysed during the current study are available in the Dryad repository, https://doi.org/10.5061/dryad.m37pvmd9g.
