# Supplementary material for: Fisheries shocks provide an opportunity to reveal multiple recruitment sources of sardine in the Sea of Japan
Source: Sci Rep. 2024 Sep 17;14:21722. doi: 10.1038/s41598-024-72925-8 (PMC11408631; doi:10.1038/s41598-024-72925-8)
Supplement: Supplementary file 1 — Supplementary Material 1 [file 41598_2024_72925_MOESM1_ESM.docx]

**Supporting Information for:**

**Title**

Fisheries shocks provide an opportunity to reveal multiple recruitment sources of sardine in the Sea of Japan

**Authors**

Tatsuya Sakamoto^1*^, Motomitsu Takahashi^1^, Kotaro Shirai^2^, Tomoya Aono^2^, Toyoho Ishimura^3, 4^

**Affiliation**

1. Fisheries Resource Research Institute, Japan Fisheries Research and Education Agency, Nagasaki, Japan

2. Atmosphere and Ocean Research Institute, The University of Tokyo, Chiba, Japan

3. Department of Chemistry and Material Engineering, National Institute of Technology, Ibaraki College, Ibaraki, Japan

4. Graduate School of Human and Environmental Studies, Kyoto University, Kyoto, Japan

*Corresponding author

Email: sakamoto.tatsuya.3p@kyoto-u.ac.jp

Address: Hakubi Center, Kyoto University

Yoshidanihonmatsucho, Kyoto Sakyo-ku, Kyoto, 606-8316, Japan

**Contents:**

Supplementary Material and Methods

Supplementary Figure S1

Supplementary Figure S2

Supplementary Figure S3

Supplementary Figure S4

Supplementary Figure S5

Supplementary Figure S6

Supplementary Figure S7

Supplementary Table S1

References

**Supplementary Material and Methods**

*Multivariate analysis of variance of otolith δ^18^O and δ^13^C for larval stage and OR_60_*

To test for the differences in otolith *δ*^18^O and *δ*^13^C for 0-60 dph and otolith radius at 60 dph among locals, nonlocals and the Pacific-offshores, multivariate analysis of variance (MANOVA) was performed, following Tabachnick et al. (2012). Data of locals and nonlocals of all 3 year-classes were pooled for MANOVA. Normality of each variable of each group was first tested using Shapiro-Wilk’s test, which showed marginally significant violations of normality in *δ*^18^O locals and Pacific-offshores (p = 0.04). However, as no significant violation for these variables in the Kolmogorov-Smirnov test (p = 0.67 for locals and 0.39 for Pacific offshores), which are more suitable for sample sizes larger than 50 (Mishra et al., 2019), we considered that the violations were negligible here. Multivariate normality was tested multivariate Shapiro-Wilk’s test, which also suggested significant violation (p = 0.0025). The Pearson’s r between the variables were between -0.26 and 0.52, suggesting limited multicollinearity. No nonlinear relationship between variables in each group was evident in the scatter plots. As homogeneity of covariances was violated (Box’s M-test, p = 3.9*10^-19^), Pillai’s multivariate statistic was used in MANOVA. Homogeneity of variance was tested by Levene’s test, which suggested that the violation in *δ*^13^C (p = 0.009), *δ*^18^O (p = 5.9*10^-6^) and otolith radius (p = 1.8*10^-6^). Because some violations were thus detected in the assumption tests as above, α = 0.01 was used as significance threshold in MANOVA to perform the test conservatively, and the non-parametric Kruskal-Wallis test and Games-Howell test were used in post-hoc tests and pairwise comparisons. These analyses were performed using R 4.1.0 with packages tidyverse 1.3,1, ggpubr 0.4.0, rstatix 0.7.0, car 3.0.11, broom 0.7.9 and GGally 2.1.2.

*Prediction of potential nursery areas of the nonlocals*

To explore the likely migration pattern of nonlocals, potential distributions during their larval and juvenile stages were inferred by comparing observed and predicted isoscape of otolith *δ*^18^O using hydrodynamic model (Aono et al., 2024). As the distributions can be either inside the SJ-ECS system or the western North Pacific, we made inferences for both patterns. To match the fish age to actual calendar dates, hatch dates of nonlocals needed to be assumed. Spawning in the SJ-ECS system peaked during April to May in 2013–2015 (Supplementary Fig. S1). The most frequently occurred hatch dates of juveniles found in the Kuroshio-Oyashio transition zone were also between mid-April and mid-May during 2013–2015 (Niino et al., 2020). Based on these observations, the mean hatch date of the nonlocals was considered to be between 16^th^ April and 16^th^ May. For calculation of isoscapes, hatch date was assumed to be every three days between 16^th^ April and 16^th^ May. For each hatch date assumption, mean temperature and salinity distributions at 10m depth during 0–60 days (larval stage) and 106–120 days (juvenile stage) from hatch were calculated using daily reanalysis data provided by the data assimilated model JADE2 (Igeta et al., 2022) for the SJ-ECS system, and FRA-ROMS for the North Pacific (Kuroda et al., 2018). From these temperature and salinity distributions, isoscape of otolith *δ*^18^O was predicted following the relationships between otolith *δ*^18^O and seawater temperature and *δ*^18^O, and between salinity and seawater *δ*^18^O specifically developed for each region:

Otolith *δ*^18^O = Seawater *δ*^18^O – 0.18*Temperature (°C) + 2.69 (Sakamoto et al., 2017),

Seawater *δ*^18^O = 0.23*Salinity – 7.54 (for the SJ-ECS system, Aono et al., 2024)

Seawater *δ*^18^O = 0.56*Salinity – 19.06 (for the Pacific, Sakamoto et al., 2019).

The model grid points of which predicted otolith *δ*^18^O was within the range of the mean ± 1 standard deviation (SD) of the analysed otolith *δ*^18^O of nonlocals of each year-class were considered as potential distributions. For visualization, the predicted distributions for each hatch date assumption were shown as translucent shades (alpha = 0.15) and overlaid in one figure using Matplotlib 3.3.4 in Python 3.8.8. To consider the possibility that the nonlocals hatched during marginal spawning months in the SJ-ECS, the distributions were also predicted assuming the hatch in every three days in February or in June. Regarding the habitat depth, Aono et al. (2024) showed that sardine juveniles leave near surface layer when temperature there is exceedingly high, which appeared to be the case in the East China Sea and southern Sea of Japan but not in the middle of Sea of Japan. As the nonlocals here had significantly higher otolith *δ*^18^O for the juvenile stage than the locals, we can assume that they had distributed in cooler regions than the middle of Sea of Japan, where sardine are likely distributed in the near surface layer.

The accuracy of the hydrodynamic model directly affects the reliability of the predictions. JADE2 (Japan Sea Data Assimilation Experiment 2) is a data assimilated hydrodynamic model aimed to reproduce realistic current field of SJ-ECS system, maintained by Fisheries Research and Education Agency (Igeta et al., 2022). This model is based on DR_M (Hirose et al., 2013), and the sea surface height and temperature available from an AVISO product, and in situ observations conducted by Japanese prefectural institutes were assimilated into the model by an approximate Kalman filtering and nudging method. The model domain is meshed by 1/12° and 1/15° in the zonal and meridional directions, respectively. Because accuracies assessments of temperature and salinity for this model is not published, we compared the modelled values to those observed by Argo floats (Supplementary Fig. S2). Argo float dataset Advanced automatic QC(AQC) Argo Data ver.1.2a distributed by JAMSTEC (Sato, 2014) was used for this comparison. The root-mean-square differences (RMSDs) between modelled and Argo float observed temperatures and salinities at depths shallower than 15m were 1.0–1.9 °C and 0.1–0.7, respectively, during February to September 2014 and 2015 (Supplementary Fig. S2). These differences corresponded to RMSD 0.2–0.3 ‰ in predicted otolith *δ*^18^O (Supplementary Fig. S2). FRA-ROMS is an ocean forecast and reanalysis system based on the Regional Ocean Modelling System (ROMS) with three-dimensional variational analysis (Kuroda et al., 2016). The aim of this system is to realistically simulate mesoscale variations in the western North Pacific, and reproduces reproduce representative features of mesoscale variations such as the position of the Kuroshio path, variability of the Kuroshio Extension, and southward intrusions of the Oyashio (Kuroda et al., 2016). The RMSDs between sea surface temperature observed by satellite and reanalysed by FRA-ROMS, estimated at monthly intervals, were in the range 0.63–1.10 °C, corresponding to 0.11–0.20‰ in otolith *δ*^18^O (Kuroda et al., 2016). The RMSDs between a monthly mean dataset of global oceanic salinity derived from Argo float observations and reanalysed salinity at 10 m depth were below 0.20 in the Kuroshio-Oyashio system, corresponding to 0.12‰ in seawater *δ*^18^O (Sakamoto et al., 2019). These assessments show that the accuracies are comparable to analytical precision of *δ*^18^O of otolith and seawater, and variability of observed otolith *δ*^18^O of the nonlocals.


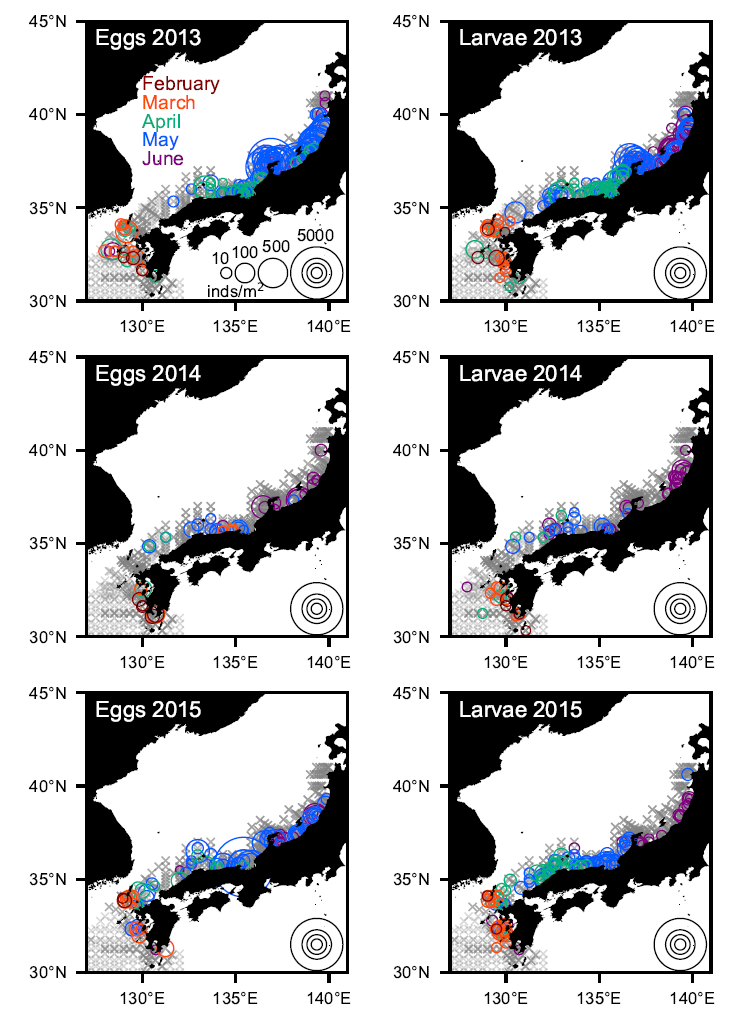
**Supplementary Figures**

**Supplementary Figure S1. Results of spawning surveys during February–June 2013–2015.** The circles and crosses show the locations where eggs or larvae were and were not found, respectively. The sizes of the circle represent the density of eggs or larvae collected by NORPAC nets. Larvae caught in the surveys are typically smaller than 7 mm.


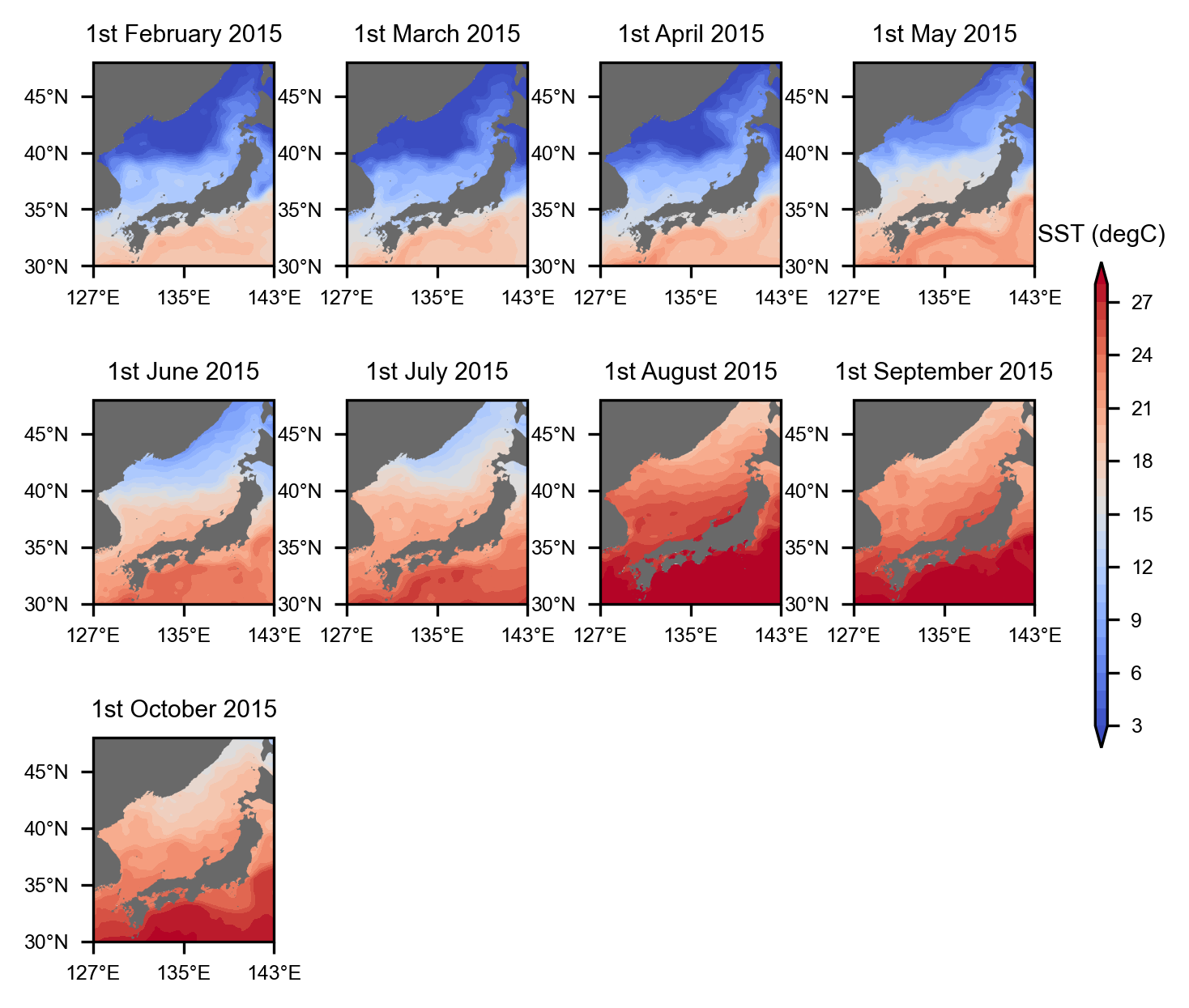


**Supplementary Figure S2. Seasonal change in sea surface temperature from 1^st^ February to 1^st^ October 2015.** Satellite based product (the operational sea surface temperature and sea ice analysis (OSTIA) system, Donlon et al., 2012) was used.


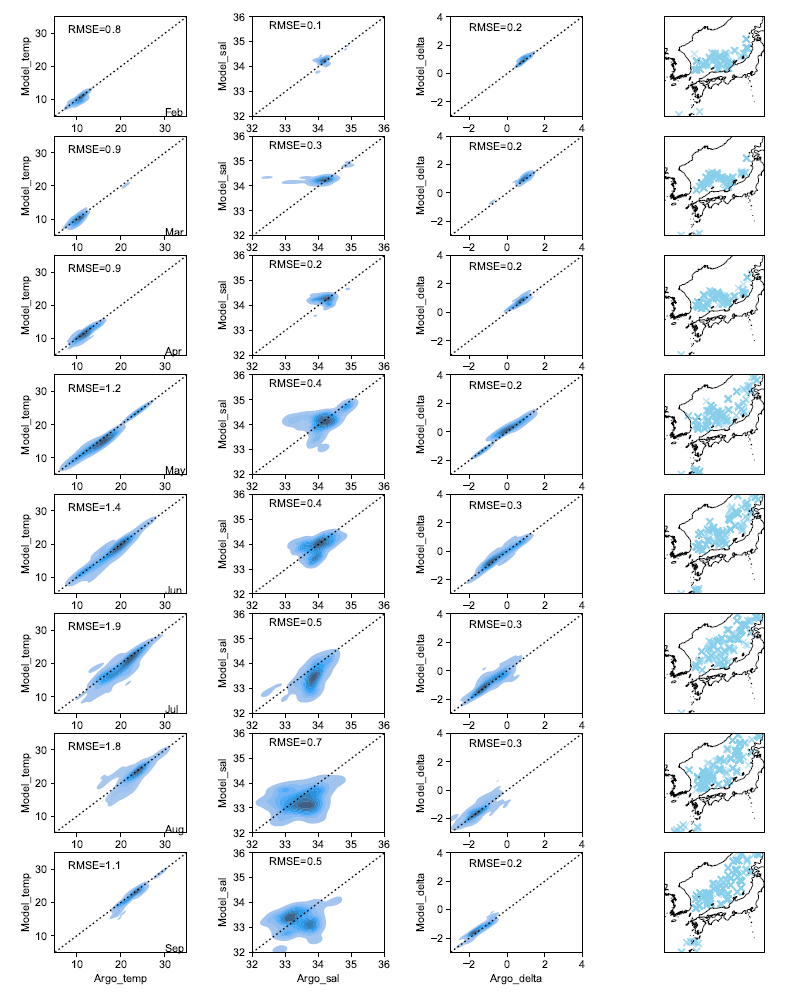


**Supplementary Figure S3. Comparison of temperature (first column) and salinity (second column) modelled by JADE-2 model and those observed by Argo floats during February–September 2014–2015.**


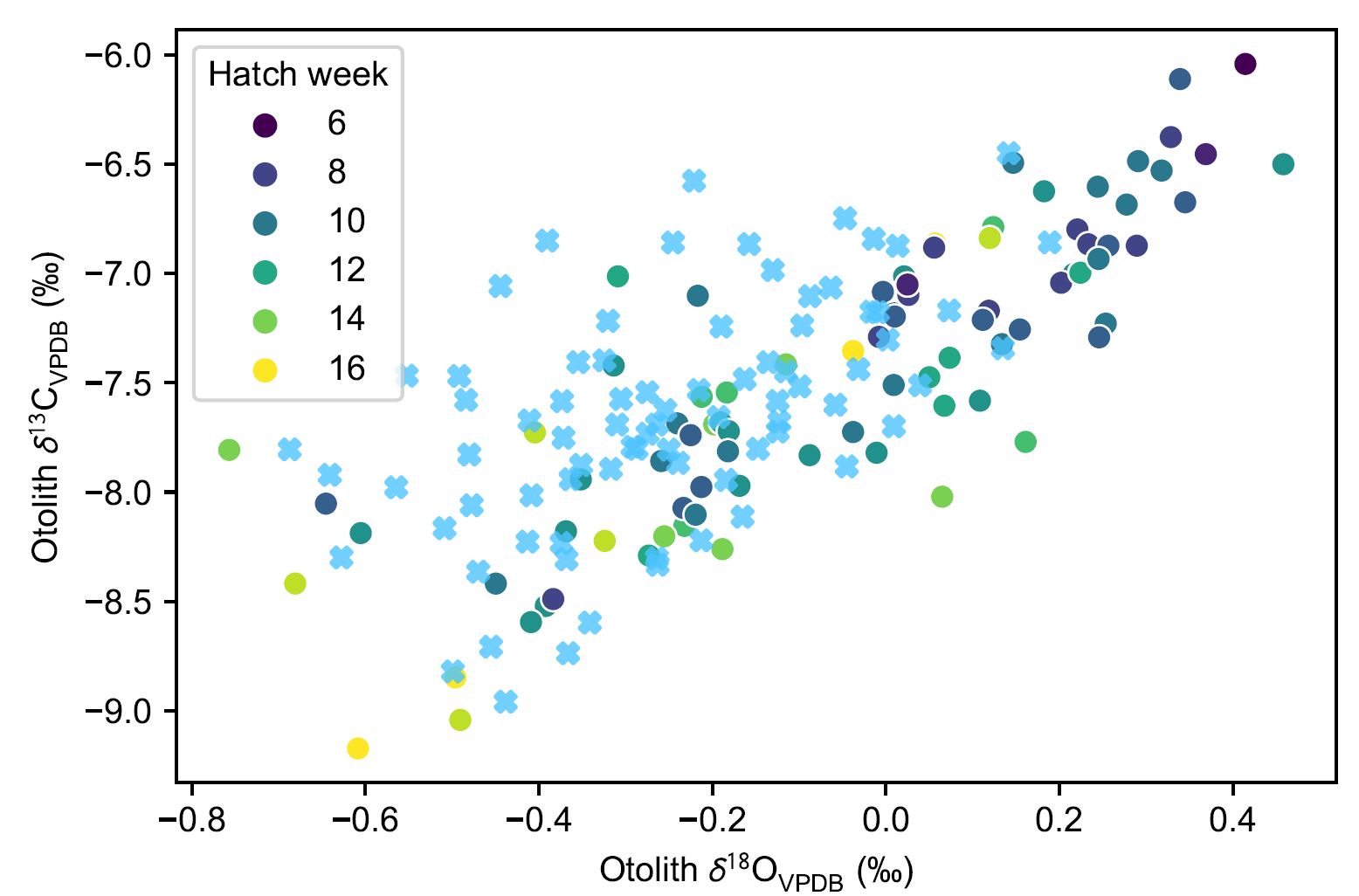


**Supplementary Figure S4. Comparison of otolith *δ*^18^O and *δ*^13^C for 0–60 dph between the nonlocals (crosses) and the pacific-offshores (circles).** The colours of the circles, the pacific-offshores, represent the week of hatch counted from January 1^st^ of each year.


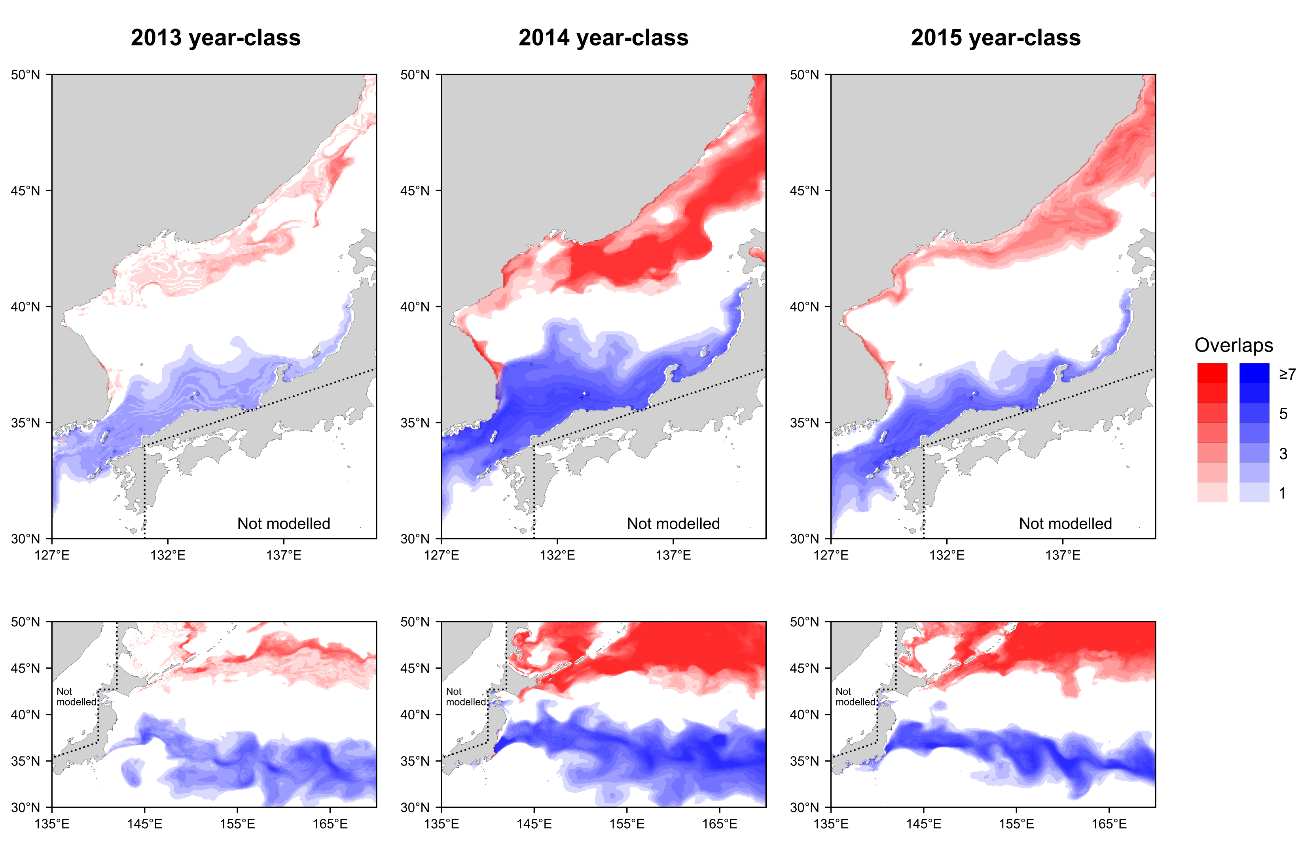


**Supplementary Figure S5. Potential distribution of nonlocals of 2013–2015 year-class during the larval (0-60 dph, blue) and juvenile (106-120 dph, red) stages in the SJ-ECS (upper row) and North Pacific (lower row) predicted based on otolith *δ*^18^O and hydrodynamic models.** The shades indicate the areas where the environmental conditions (temperature and salinity) would produce an otolith *δ*^18^O within 1 SD of the mean value for the nonlocals. Darker shades indicate the areas where the environmental conditions match for multiple hatch dates within the range tested (every 3 days between mid-April and mid-May). Dotted lines suggest the boundary of the modelled area.

**
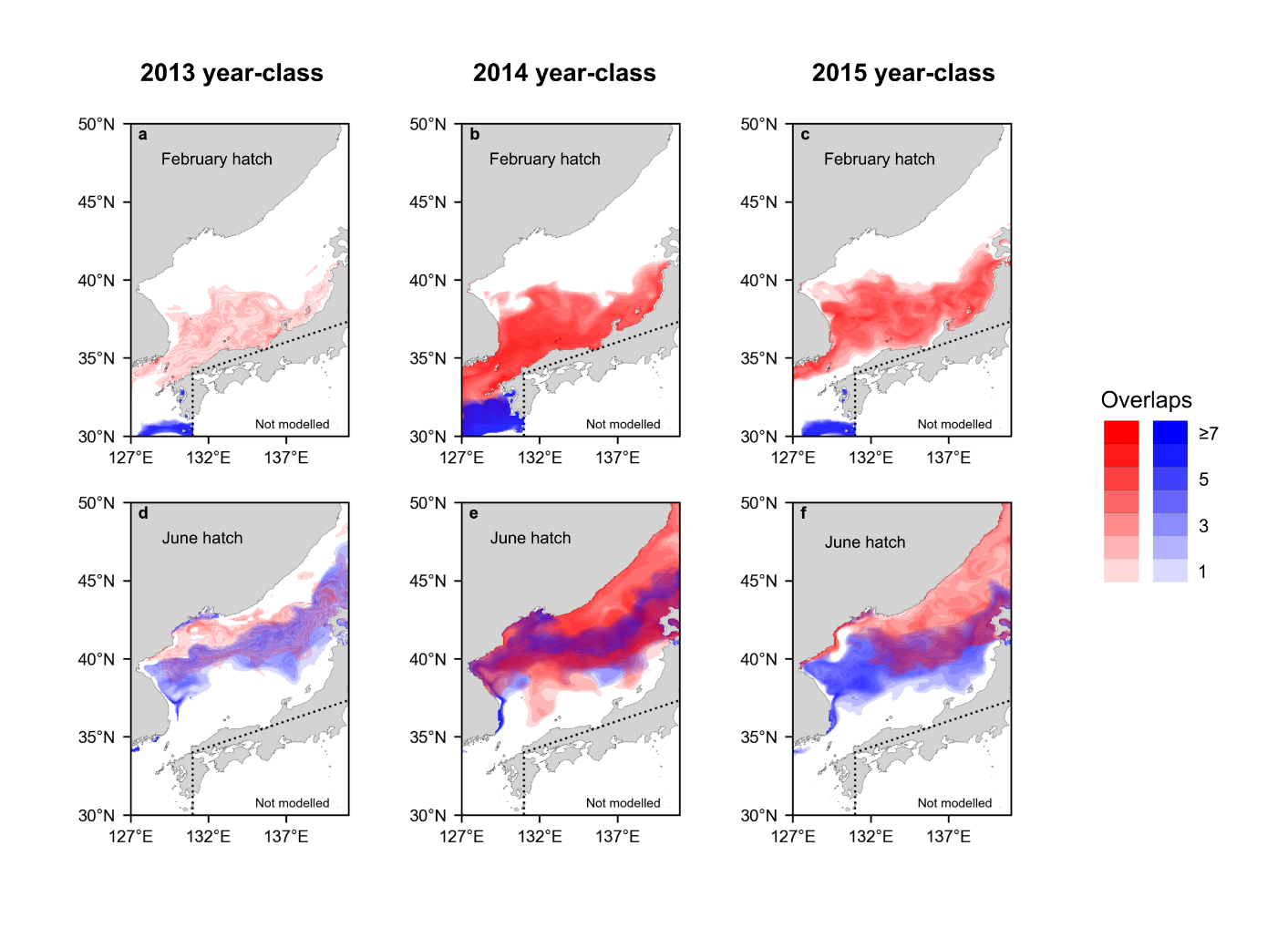
**

**Supplementary Figure S6. Potential distribution of nonlocals of 2013–2015 year-class during the larval (0-60 dph, blue) and juvenile (106-120 dph, red) stages in the SJ-ECS assuming hatch dates in February (a–c) and in June (d–f).** The shades indicate the areas where the environmental conditions (temperature and salinity) would produce an otolith *δ*^18^O within 1 SD of the mean value for the nonlocals. Darker shades indicate the areas where the environmental conditions match for multiple hatch dates within the range tested [every 3 days in February (a–c) and in June (d–f)]. Dotted lines suggest the boundary of the modelled area.


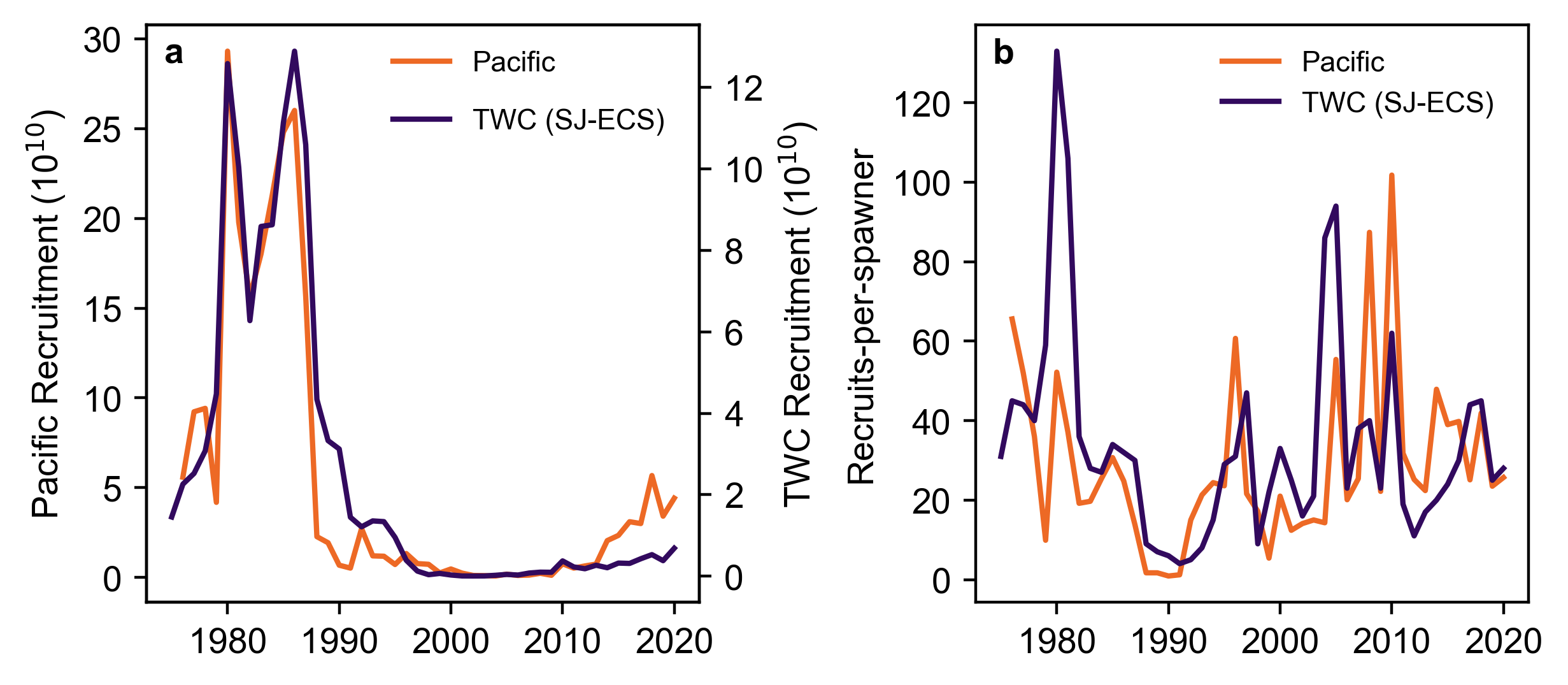


**Supplementary Figure S7. Timeseries of recruitment (a) and recruits-per-spawner (b) of Pacific (orange) and Tsushima Warm Current (purple) stocks estimated by stock assessment models for each.**

**Supplementary Table S1. Results of pairwise comparisons of otolith *δ*^18^O and *δ*^13^C values for 0-60 dph and otolith radius at 60 dph between the locals, nonlocals and Pacific-offshores based on Games-Howell test.**

**References**

T. Aono, T. Sakamoto, T. Ishimura, M. Takahashi, T. Yasuda, S. Kitajima, K. Nishida, T. Matsuura, A. Ikari, S. Ito, Migration patterns of the Japanese sardine in the Sea of Japan by combining the microscale stable isotope analysis of otoliths and an ocean data assimilation model. *Frontiers in Marine Science.***11**, (2024).

C. J. Donlon, M. Martin, J. Stark, J. Roberts-Jones, E. Fiedler, W. Wimmer, The operational sea surface temperature and sea ice analysis (OSTIA) system. *Remote Sens. Environ.***116**, 140-158 (2012).

N. Hirose, K. Takayama, J-H Moon, T. Watanabe, T. Nishida T, Regional data assimilation system extended to the East Asian Marginal Seas. *Umi Sora (Sea and Sky)*, **89**, 1–9 (2013). doi: info:ndljp/pid/10116140

Y. Igeta, C. Sassa, M. Takahashi, M. Kuga, S. Kitajima, T. Wagawa, S. Abe, C. Watanabe, T. Setou, H. Nakamura, N. Hirose, Effect of interannual variations of Kuroshio–Tsushima Warm Current system on the transportation of juvenile Japanese jack mackerel (Trachurus japonicus) to the Pacific coast of Japan. *Fisheries Oceanography.***32**, 133-146 (2023).

H. Kuroda, T. Setou, S. Kakehi, S. Ito, T. Taneda, T. Azumaya, D. Inagake, Y. Hiroe, K. Morinaga, M. Okazaki, Recent Advances in Japanese Fisheries Science in the Kuroshio-Oyashio Region through Development of the FRA-ROMS Ocean Forecast System: Overview of the Reproducibility of Reanalysis Products. *Open Journal of Marine Science.***7**, 62 (2016).

P. Mishra, C. M. Pandey, U. Singh, A. Gupta, C. Sahu, A. Keshri, Descriptive statistics and normality tests for statistical data. *Annals of Cardiac Anaesthesia.***22**, 67-72 (2019).

Y. Niino, S. Furuichi, Y. Kamimura, R. Yukami, Spatiotemporal spawning patterns and early growth of Japanese sardine in the western North Pacific during the recent stock increase. *Fish. Oceanogr.***30**, 643-652 (2021).

T. Sakamoto, K. Komatsu, K. Shirai, T. Higuchi, T. Ishimura, T. Setou, Y. Kamimura, C. Watanabe, A. Kawabata, Combining microvolume isotope analysis and numerical simulation to reproduce fish migration history. *Methods Ecol Evol.***10**, 59-69 (2019).

T. Sakamoto, K. Komatsu, M. Yoneda, T. Ishimura, T. Higuchi, K. Shirai, Y. Kamimura, C. Watanabe, A. Kawabata, Temperature dependence of *δ*^18^O in otolith of juvenile Japanese sardine: Laboratory rearing experiment with micro-scale analysis. *Fisheries Research.***194**, 55-59 (2017).

K. Sato. Advanced automatic QC Argo Data. *JAMSTEC*. (2014). doi:10.17596/0000104
